# Feasibility of a tailored operative strategy from organ preservation to pelvic exenteration for cT4 rectal cancer depending on neoadjuvant response

**DOI:** 10.1007/s00384-024-04675-y

**Published:** 2024-07-31

**Authors:** Christina Fleming, Deena Harji, Benjamin Fernandez, Marc-Olivier François, Vincent Assenat, Pasticier Gilles, Michiels Clément, Grégoire Robert, Quentin Denost

**Affiliations:** 1 Bordeaux Colorectal Institute, Clinique Tivoli, 220 Rue Mandron, 33000 Bordeaux, France; 2 Department of Digestive Surgery, Colorectal Unit, Haut-Lévêque Hospital, Bordeaux University Hospital, Pessac, France; 3 Royal College of Surgeons in Ireland, Dublin, Ireland; 4 Department of Urologic Surgery, Clinique Tivoli, Bordeaux, France; 5 Department of Urologic Surgery, CHU Bordeaux University Hospital, Bordeaux, France

**Keywords:** Rectal cancer, Organ preservation, Pelvic exenteration, Total neoadjuvant therapy

## Abstract

**Purpose:**

Improvements in neoadjuvant therapy for locally advanced cT4 rectal cancer have led to improved tumour response and thus a variety of suitable management strategies. The aim of this study was to report management and outcomes of patients with cT4 rectal cancer undergoing a spectrum of treatment strategies from organ preservation (OP) to pelvic exenteration (PE).

**Methods:**

Patients who underwent elective treatment for cT4 rectal cancer between 2016 and 2021 were included. All patients were treated with curative intent. Surgical management was adapted to tumour response. Kaplan–Meier curves were generated to compare 3-year overall survival (3y-OS), local recurrence (3y-LR) and distant metastases (3y-DM) between different strategies.

**Results:**

Among 152 patients included, 13 (8%) underwent OP, 71 (47%) TME and 68 (45%) APR/PE. The median follow-up was 31.3 months. Patients undergoing OP had a lower tumour pretreatment (*p* < 0.001). Compared to patients with TME, those with APR/PE had a higher rate of ypT4 (*p* = 0.001) with a lower R0 rate (*p* = 0.044). The 3y-OS and 3y-DM were 78% and 15.1%, respectively, without significant differences. The 3y-LR was 6.6%, and patients with OP had a significantly worse 3y-local regrowth compared to 3y-LR in patients with TME and APR/PE (30.2% vs. 5.4% vs. 2%, *p* = 0.008).

**Conclusion:**

cT4 tumours may be suitable for the full spectrum of rectal cancer management from organ preservation to pelvic exenteration depending on tumour response to neoadjuvant therapy. However, careful attention is required in OP as local regrowth in up to 30% of cases reinforces the need for sustained active surveillance in Watch&Wait programmes.

## Introduction

Rectal cancer accounts for approximately 35% of total colorectal cancers [1]. Up to 20% of patients with rectal cancer present with locally advanced disease (T4), including peritoneal involvement (T4a) or invasion into adjacent organs (T4b) at diagnosis [2]. Historically, patients with locally advanced T4 rectal cancers involving adjacent organs requiring pelvic exenteration (PE) were offered surgery alone or were considered not suitable for surgical resection with curative intent due to the extent of the tumour [3–5]. Neoadjuvant treatment can facilitate improved surgical resection rates by downsizing the tumor to facilitate a clear surgical margin (R0) [6, 7]. Long-term survival in rectal cancer has been considerably improved by multimodal treatments including standardization of surgical technique and improvement in perioperative care, systemic chemotherapy and/or chemoradiotherapy have all conferred oncological benefit [7–12].

A wide variety of surgical approaches can be offered in modern rectal cancer management depending on tumour response following neoadjuvant therapy. Total mesorectal excision (TME) is long established as the optimum standardised operative approach to rectal cancer surgery [13]. Large tumors with threatened circumferential resection margins (CRM) and in which nodal involvement is suspected are usually treated with a combination of preoperative neoadjuvant chemoradiotherapy (NCART) and TME, which decreases the risk of a local recurrence [6, 14]. In more recent times, neoadjuvant chemotherapy is also added to this therapeutic strategy as total neoadjuvant therapy (TNT) with higher rates of tumour response and complete tumour response observed [15]. With persistent tumour invasion into adjacent organs following neoadjuvant treatment, extensive surgery with en bloc resection of other pelvic organs in the form of pelvic exenteration (PE) can still offer curative oncological resection [16]. If a good response to neoadjuvant therapy (in the form of a complete or near complete clinical response) is achieved, alternative and less extensive surgical options may be feasible including conversion from PE to TME or, in best case scenario, local excision or direct to Watch & Wait (W&W) organ preservation (OP) [17, 18]. Complete omission of surgery with direct enrollment into a W&W programme for those who achieve a complete clinical response (cCR) is becoming increasingly acceptable and practiced [19, 20].

The aim of this study was to analyse and report oncological outcomes of consecutive patients with cT4 rectal cancer undergoing the full spectrum of rectal cancer surgical management (from organ preservation to pelvic exenteration) based on response to neoadjuvant treatment.

## Materials and methods

### Patient selection

Consecutive patients who underwent elective treatment for cT4 rectal cancer over a 5-year period (January 2016 to April 2021) in a high-volume rectal cancer center were retrospectively identified in a prospectively maintained database. All patients were confirmed as cT4 on staging pelvic magnetic resonance imaging (MRI) performed at the time of diagnosis. Patients who died within 90 days of surgery were excluded due to lack of medium to long-term follow-up. Institutional Review Board approval was prospectively obtained.

### Staging and neoadjuvant therapy

All patients underwent preoperative evaluation and cancer staging, including colonoscopy with tumor biopsy to confirm diagnosis and a computed tomography thoraco-abdomino-pelvic (CT TAP) scan to rule out distant metastases. A pelvic MRI was performed for local rectal cancer staging. Liver MRI was systematically performed in cases where synchronous liver lesions concerning for metastases were identified on staging CT TAP. All patients were discussed at a dedicated multidisciplinary colorectal cancer meeting. Neoadjuvant long-course chemoradiation therapy was delivered as 45–50.4 Gy delivered in daily fractions of 1.8–2 Gy over a 5- to 6-week period combined with 5-fluorouracil [5-FU] or capecitabine [Xeloda]). Induction chemotherapy before chemoradiation was also delivered, where indicated, as FOLFOX or FOLFIRINOX for 4–6 cycles. If a complete clinical response (cCR) was achieved after neoadjuvant treatment, organ preservation (with a structured watch and wait strategy) was offered. Patients followed by watch and wait were monitored every 4 months with: digital rectal examination, endorectal ultrasonography and pelvic MRI and 6 monthly CT TAP.

### Surgical approach

Surgery was performed at a minimum of 8 weeks after the completion of chemo-radiotherapy and further improved response was not expected. Patients demonstrating a favourable response at this point were followed as part of a defined Response Surveillance Programme (RSP) [21]. All included patients were operated on with a curative intent and cases with metastases were excluded. In cases of near-complete response with tumour scar ≤ 2 cm, a Local Excision (LE) was considered. LE was performed as a traditional or endoscopic full thickness transanal rectal wall excision with a margin > 1 mm. Total mesorectal excision (TME) was performed in cases of mid or low rectal cancer with anticipated tumor-free resection margins. Abdominoperineal resection (APR) was performed in cases of intersphincteric space, external anal-sphincter and/or levator-ani muscle invasion [22] and pelvic exenteration when the tumor was invading adjacent organs (sacrum posteriorly and genitourinary organs anteriorly).

### Definitions

The classification of cT4 was defined by a primary rectal cancer involving or abutting adjacent organs or structures and defined as cT4 based on the first staging pelvic MRI performed at diagnosis. Postoperative complications were defined as any adverse event that occurred within 30 days following surgery. These were reported according to the Clavien-Dindo (CD) classification. Severe complications were defined as grade CD III or IV. Post-operative mortality was defined as death from any cause within 30 days of the resection or during the same hospital stay. Post-operatively, patients were reviewed in the outpatient department 1 month after surgery then every 4 months for the first 2 years, and every 6 months thereafter. CT TAP and serum carcinoembryonic antigen (CEA) levels were routinely performed during each follow-up visit. A full colonoscopy was performed 2 years after surgery and then once every 4 years. If recurrence was suspected, MRI and/or positron emission tomography-CT were used to confirm the diagnosis. Biopsy was selectively performed as indicated. Overall survival (OS) was calculated from time of surgery to the last follow-up visit or death for any reason. Local and distant recurrence were defined as the time from surgery to the date of first clinical or radiological diagnosis of tumor recurrence.

### Statistical analysis

For analysis, patients with APR and PE were pooled together as both groups represent poor responders to neoadjuvant treatment. Categorical variables were expressed as number and percentage. Continuous variables were expressed as median and range. Chi-squared test was used to compare categorical variables, and a non-parametric Mann–Whitney *U* test to compare continuous variables. All tests were two-sided, with type I error set at α = 0.05. Kaplan–Meier was used to estimate recurrence rate and survival.

## Results

### Patient’s characteristics

A total of 152 patients with cT4 rectal cancer were treated during the study period. Demographics and clinical characteristics are summarized in Table 1. Patients in OP group had a low rectal cancer in 100% of cases, compared to 28% and 74% (*p* < 0.001) in TME and APR/PE groups, respectively. The tumour size in OP was 5 (3–6) cm, significantly smaller than in the two other groups (*p* < 0.001). At presentation, 48 (32%) patients had synchronous metastases. Regarding neoadjuvant treatment, there was no difference between groups. 64 (42%) patients received preoperative chemo-radiotherapy, 62 (41%) induction chemotherapy followed by chemo-radiotherapy and 8 (5%) patients respectively chemotherapy or radiotherapy alone. Short course radiotherapy was delivered for 5 (3%) patients.

**Table 1:** Patient’s characteristics

	OP	TME	APR/PE	*p*
	*N* = 13	*n* = 71	*n* = 68	
Gender				0.063
Male	9 (69%)	52 (73%)	37 (54%)	
Female	4 (31%)	19 (27%)	31 (46%)	
Age (years)*	65 (39–72)	63 (32–90)	65 (37–89)	0.424
ASA score**				0.272
1	5 (56%)	17 (24%)	21 (32%)	
2	4 (44%)	41 (57%)	36 (54%)	
3	0	13 (18%)	9 (14%)	
Height from anal verge (cm)*	2 (1–4.5)	8 (1–15)	3 (1–13)	** < 0.001**
Low	13 (100%)	20 (28%)	50 (74%)	** < 0.001**
Mild	0	39 (55%)	13 (19%)	
High	0	12 (17%)	5 (7%)	
cN				0.114
+	9 (69%)	64 (90%)	56 (82%)	
0	4 (31%)	7 (10%)	56 (82%)	
Metastasis				0.430
Yes	3 (23%)	26 (37%)	19 (28%)	
No	10 (77%)	45 (63%)	49 (72%)	
Tumour size (cm)*	5 (3–6)	6 (2–11)	7 (2.3–12.6)	** < 0.001**
Neoadjuvant treatment				0.503
Yes	13 (100%)	65 (92%)	64 (94%)	
No	0	6 (8%)	4 (6%)	

*Median (range), **missing data

### Surgical approach and operative outcomes

Surgical approach and operative outcome data are summarized in Table 2. OP strategy was performed in 9% (*n* = 13). Within this groups 12 patients (93%) went direct to Watch-and-Wait and 1 (7%) underwent LE prior. TME was performed in 71 (47%) patients, and APR or PE in 68 (45%) patients. Surgery was performed laparoscopically in 65 (46%) patients, robotically in 26 (19%) patients and by open approach in 48 (34%) patients. Twenty-nine (21%) patients experienced severe postoperative complications (Dindo ≥ III) and the median length of hospital stay was 9 days (1–97). A minimally invasive approach was used significantly more often in TME (*p* = 0.002) with a lower rate of post-operative morbidity (*p* = 0.044) and a shorter length of hospital stay (*p* < 0.001) compared to APR/PE.

**Table 2:** Intraoperative data and specimen analysis (OP excluded)

	TME	APR/PE	*p*
	*n* = 71	*n* = 68	
Surgical approach			**0.002**
Laparoscopic	42 (59%)	23 (34%)	
Robotic	14 (20%)	12 (18%)	
Open	15 (21%)	33 (48%)	
Surgical morbidity			**0.044**
Dindo 0-I-II	61 (86%)	49 (72%)	
Dindo III-IV	10 (14%)	19 (28%)	
Hospital stay (days)*	7 (2–28)	12 (4–97)	< **0.001**
pT			**0.001**
pT0	5 (7%)	3 (4%)	
pT1	2 (3%)	0	
pT2	6 (9%)	5 (7%)	
pT3	49 (69%)	30 (44%)	
pT4	9 (13%)	30 (44%)	
pN†			0.253
pN0	33 (47%)	41 (60%)	
pN1	24 (34%)	18 (27%)	
pN2	14 (20%)	9 (13%)	
Vascular invasion			0.100
Yes	37 (52%)	26 (38%)	
No	34 (48%)	42 (62%)	
Neural invasion			0.730
Yes	26 (37%)	23 (34%)	
No	45 (63%)	45 (66%)	
Resection			**0.044**
R0	61 (86%)	49 (72%)	
R1	10 (14%)	19 (28%)	

*Median (range)

### Pathological outcomes

On final pathological analysis, 118 (85%) tumours were ypT3-T4 with a significantly lower rate of ypT4 in the TME group compared to APR/PE group (13% *vs.* 44%, *p* = 0.001). A complete tumour response (ypT0) was observed in 8 (6%) patients. A clear R0 resection margin was achieved in 79% of cases (*n* = 110) and more frequently in the TME group (86% *vs.* 72%, *p* = 0.044). Positive lymph nodes were identified in 65 (47%) patients, without significant difference between groups and similar for vascular and neural invasion.

### Impact of surgical strategy on long-term outcome

**Fig. 1:**
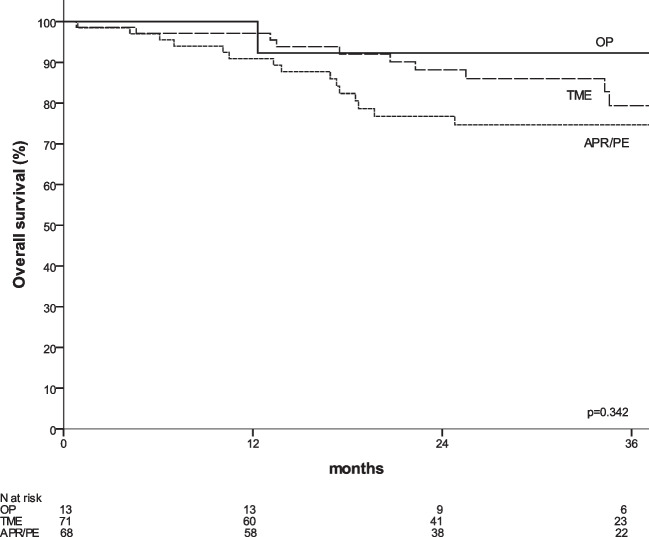
Overall survival according to surgical procedure

**Fig. 2:**
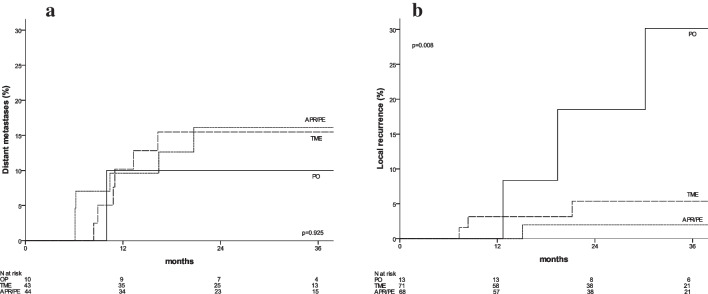
**A** Distant metastases according to surgical procedure. **B** Local recurrence according to surgical procedure

The median follow-up was 31.3 months (12.3–54.1), 29.0 months (0.8–65.1) and 25.1 months (0.3–58.4) respectively for organ preservation, TME, APR/PE group (*p* = 0.636). Subgroup analysis according to surgical procedure are summarized in Table 3. The overall 3y-OS was 78%, without significant different between OP, TME and APR/PE (92% *vs.* 79% *vs.* 75%, *p* = 0.342) (Fig. 1). The 3y-DM was 15.1%, without significant different between OP, TME and APR/PE (10% *vs.* 15.5% *vs.* 16.1%, *p* = 0.925) (Fig. 2). The 3y-LR was 6.6%, patients with OP had a significantly higher 3y-local regrowth compared to 3y-LR in patients with TME and APR/PE (30.2% *vs.* 5.4% *vs.* 2%, *p* = 0.008) (Table 3). Local regrowth in the OP group were treated by local excisions (n = 2) and APR (*n* = 1).

**Table 3:** Oncological follow-up according surgical procedures

	Overall study	OP	TME	APR/PE	*p*-value
	(*n* = 152)	(*n* = 13)	(*n* = 71)	(*n* = 68)	
Follow-up (months)*	27.4	31.3	29.0	25.1	0.636
	(0.3–65.1)	(12.3–54.1)	(0.8–65.1)	(0.3–58.4)	
Overall survival					0.342
At 1 year	95%	100%	97%	91%	
At 3 years	78%	92%	79%	75%	
Local recurrence					**0.008**
At 1 year	1.4%	0%	3.2%	0%	
At 3 years	6.6%	30.2%	5.4%	2.0%	
	(*n* = 97)	(*n* = 10)	(*n* = 43)	(*n* = 44)	
Distant metastases					0.925
At 1 year	10.0%	10.0%	10.2%	9.6%	
At 3 years	15.1%	10.0%	15.5%	16.1%

## Discussion

In this study, clinical and oncological outcomes of patients with cT4 rectal cancer undergoing the full spectrum of rectal cancer surgical management from organ preservation (OP) to pelvic exenteration (PE) have been reported. In this tailored approach to cT4 management guided by tumour response following neoadjuvant therapy, the 3y-OS, 3y-DM and 3y-LR rates were 78%, 15.1% and 6.6%, respectively. This can be considered a therapeutic success in this group of high-risk patients. However, the 3y-local regrowth was significantly higher in patients with OP compared to overall 3y-LR rates, which is not unexpected and highlights the essential need for sustained surveillance in W&W programmes when OP is considered suitable. Patient selection was suitable considering the low 6% ypT0N0 rate in final pathology of the resectional groups and over 40% of patients in the APR/PE group remained ypT4.

cT4 tumours represent a complex and high-risk rectal cancer patient population with significant concern for CRM positivity and poorer oncological outcomes. Total neoadjuvant therapy (TNT) in this setting, which involves the additional administration of preoperative systemic chemotherapy in combination with chemoradiotherapy before surgery, has demonstrated favourable results and is being adopted clinically [23–27]. In France, induction chemotherapy is generally preferred in TNT compared to consolidation chemotherapy [8, 28]. The hypothesis is that systemic chemotherapy targets circulating micrometastases reducing the opportunity for such to mestastasise [24]. Addition of chemotherapy further increases the opportunity to develop a complete clinical response (cCR), even in cT4 tumours and increase the opportunity to consider organ preservation (OP) [24]. Therefore, we are now in an era where a complete spectrum of management from organ preservation to pelvic exenteration can be considered for cT4 tumours as is outlined in this study.

Adoption of OP for cCR in rectal cancer is increasing [18–21]. In few situations, cT4 tumours develop a complete response; however, many W&W programmes tend to selectively enroll lower cT stage tumours [18–20]. In our practice, an opportunistic approach to patient selection is followed thus any cT tumours are considered if cCR is achieved [21]. In this study, the OP group all had low tumours (median distance of 2 cm from the anal verge) and as they were, cT4 would have required an APR for a resectional approach due to sphincter complex involvement from the outset if cCR hadn’t occurred. In the OP group, tumours were slightly smaller (5 cm vs 6 and 7 cm) compared to the other two groups and all bar one achieved cCR with only one patient in this group requiring a local excision. As local regrowth occurred in 30% of the group within 3 years of follow-up, active surveillance as part of a W&W programme is crucial, particularly in higher locally advanced tumour stages where the risk of regrowth is higher [29]. Also, as local regrowth can be associated with an increased risk of distant metastases, early identification and rapid management of local regrowth are essential [29]. This finding was not reproduced in this study, likely due to the small number of included patients in the OP group (*n* = 13) and also early re-operation (within 3 weeks of identification) when local regrowth was identified [21].

As expected, in cT4 rectal cancer, the risk of CRM positivity is high and was the initial rationale for neoadjuvant therapy. The R1 resection rate in this cohort was 21% with the highest rate (28%) observed in the APR/PE group where the post-treatment ypT stage remained as ypT4 in over 40% of patients. This R1 rate is in keeping with previous contemporary data which broadly reports R1 resection rates ranging from 13 to 31.6% in cT4 colorectal cancer [30–32]. As these reported cohorts represented both colon and rectal cancers, it would be expected that the R1 rectal cancer rate specifically may be higher. CRM status is considered one of the most significant factor in the long-term outcomes of patients with rectal cancer [33]. Pelvic MRI is considered as the gold standard investigation to pre-operatively identify patients at high risk of a positive CRM [34]. Multiple studies have shown that TME with negative CRM is associated with decreased local recurrence and improved disease-free survival following rectal resection [35, 36]. Therefore, beyond TME, surgery is required to obtain an R0 resection for locally advanced disease in certain circumstances where a clear CRM cannot be achieved by standard TME. Of note, the higher R1 rate in the APR/PE group in this study was not associated with an increased 3y-LR (2% vs 5.4%).

The overall survival rate for all cT4 groups in this study was 78% at 3 years, which is comparable to, or even better than, rates among patients with colorectal cancer in general in two population-based studies [37, 38]. In this cohort, 3y-OS was 92% in those who achieved a cCR and were managed by OP. This result is equivalent to previous data published in meta-analysis [39]. The risk of local regrowth was similar to results of patients managed with W&W from all clinical rectal cancer stage combined, i.e. cT1-cT4 [40]. The authors therefore conclude that the oncological prognosis for patients with initially cT4 rectal cancer who underwent OP after cCR seems to be equivalent to long-term survival outcome of non-locally advanced rectal cancer. If OP is proposed for cT4 rectal cancer who opportunistically develop a cCR, the high risk of local regrowth during W&W follow-up must be communicated to patients and the associated increased risk of distant metastasis for up to 5 years after the incidence of regrowth [41].

In this study, patients treated by TME had a worse 3y-OS compared to the W&W group (80% *versus* 92%) but the recurrence-free survival rate was similar. Most mortalities were in patients for whom a distant recurrence (metastases) was diagnosed after TME surgery for initial cT4 rectal cancer. Overall, there was a higher rate of distant metastasis in the TME (15.5%) and APR/PE (16.1%) groups compared to OP group (10%). This may be due to tumour biology and tumour response to chemoradiotherapy [24]. The ability to systematically treat and control tumour micrometastases may have resulted in less distant metastases in the ‘best’ responders who were managed by OP compared to poorer responders who required full cancer resections. Notably, 50% of patients who developed local regrowth after W&W did develop distant metastases but this was only one patient as the OP cohort was so small. The APR/PE had a better 3y-LR further supporting the role for PE in LARC where CRM is threatened.

There are limitations to this study that require discussion. This analysis was performed retrospectively and thus the associated methodological limitations exist. It is also a review of single institution practice in a specialized high volume rectal cancer practice, therefore the generalizability of the findings should be considered in this context. A variety of neoadjuvant therapy regimen were also utilized which may impact the oncological outcome and overall survival, however as patients were managed based on their tumour response to neoadjuvant therapy, this effect should have been minimized.

In conclusion, cT4 locally advanced rectal cancer tumours are a complex, high risk cancer group but as demonstrated from this data are suitable for a wide spectrum of management strategies from organ preservation to pelvic exenteration depending on tumour response to neoadjuvant therapy. Regardless of operative strategy, favourable and similar oncological outcomes were demonstrated when all surgical options were practiced and patients appropriately selected. Sustained active surveillance of a W&W programme is required as local regrowth rate in cT4 tumours can be as high as 30% at 3 years.

## Data Availability

Primary data used for analysis this study can be made available from the Principe investigator following reasonable request.
